# Kinetic Mechanism of Substoichiometric Inhibition of Huntingtin Exon‐1 Protein Aggregation by Selenium Nanoparticles

**DOI:** 10.1002/smsc.202500345

**Published:** 2025-09-13

**Authors:** Francesco Torricella, Vitali Tugarinov, G. Marius Clore

**Affiliations:** ^1^ Laboratory of Chemical Physics National Institute of Diabetes and Digestive and Kidney Diseases National Institutes of Health Bethesda MD 20892‐0520 USA; ^2^ Present address: Brain and Development Disease Laboratory Istituto Italiano di Tecnologia Via Morego 30 16163 Genova Italy

**Keywords:** Huntington's disease, inhibition of fibril formation, protein aggregation, selenium nanoparticles

## Abstract

Accumulation of huntingtin exon‐1 protein (htt^ex1^) fibrils within neurons occurs when the polyglutamine region exceeds ≈35 residues and is responsible for Huntington disease, a fatal neurodegenerative condition. Recent work has shown that selenium nanoparticles (SeNP) are protective against neurodegeneration. Herein, the mechanistic basis for SeNP modulation of htt^ex1^ aggregation is explored. Fibril formation of htt^ex1^ entails two distinct processes on timescales differing by many orders of magnitude: prenucleation oligomerization on the microsecond timescale to generate a low population of transient tetramers that undergo slow (hours timescale) unimolecular conversion into elongation‐competent nuclei, followed by elongation and secondary nucleation. Using NMR spectroscopy, fluorescence immunostaining, and transmission electron microscopy, the interaction of SeNPs with two htt^ex1^ protein constructs, htt^ex1^Q_7_ and htt^ex1^Q_35_ containing 7 and 35 glutamine repeats, respectively, is studied. htt^ex1^Q_7_ undergoes transient prenucleation tetramerization but remains largely monomeric over a period of weeks, while htt^ex1^Q_35_ forms fibrils within a period of hours. It is shown that SeNPs reduce the rate of fibril formation substoichiometrically with respect to monomer by selectively targeting and binding with nanomolar affinity to the extendable ends of elongation‐competent species of htt^ex1^Q_35_, thereby reducing the pool of free extendable ends.

## Graphical abstract

Using a combination of NMR spectroscopy, fluorescence immunostaining, and electron microscopy, it is shown that selenium nanoparticles reduce the rate of huntingtin exon‐1 protein fibril formation, substoichiometrically with respect to monomer, by targeting and binding with nanomolar affinity to the extendable ends of elongation‐competent species formed along the aggregation pathway, thereby reducing the pool of free extendable ends.

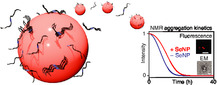

## 1 Introduction

**Figure 1:**
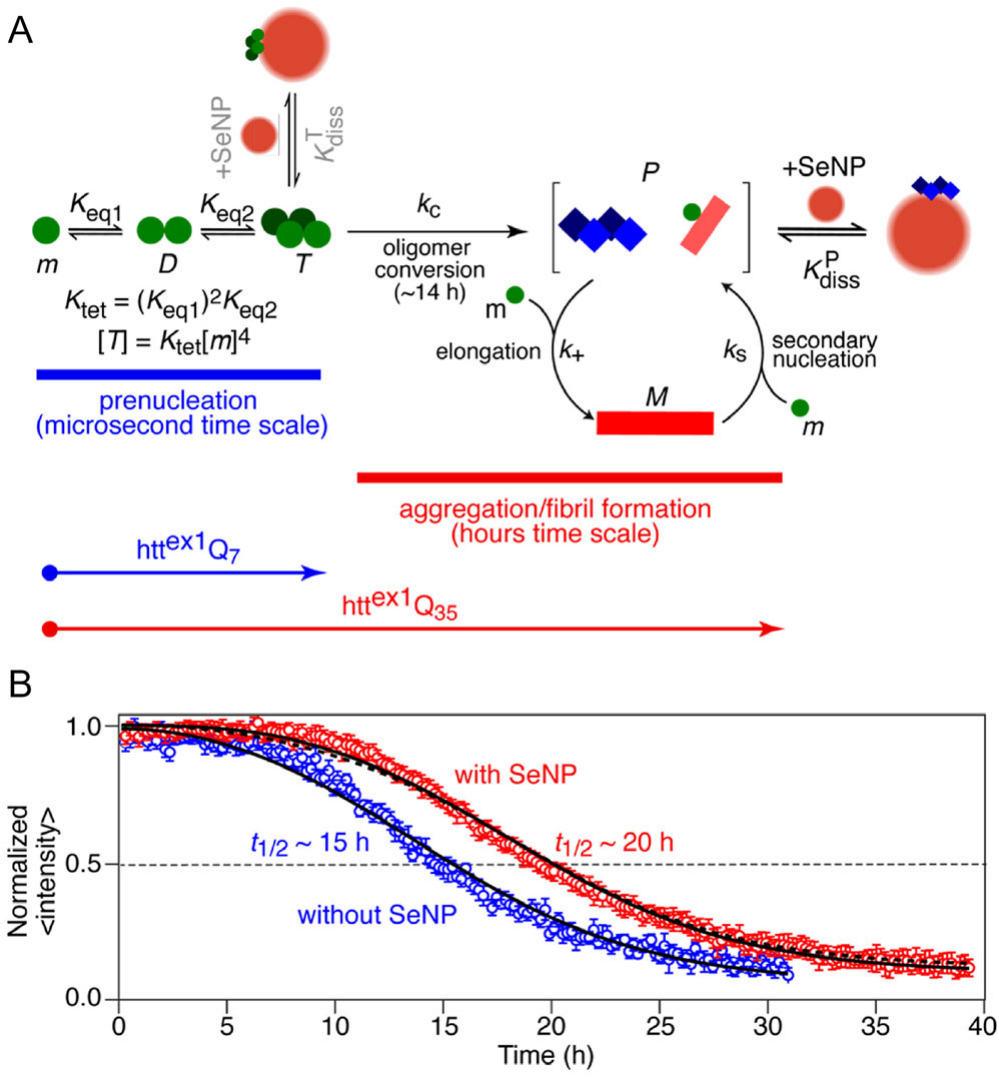
Effect of SeNPs on the aggregation/fibrillization of htt^ex1^Q_35_. A) Schematic of the aggregation mechanism of htt^ex1^Q_35_ (see text Section 1). The SeNPs bind with nanomolar affinity to the ends *P*. B) Aggregation kinetics at 5 °C and pH 6.5 of 300 μM htt^ex1^Q_35_ in the absence and presence of 8 mg ml^−1^ (42 nM) SeNPs^[^ ^50^ ^]^ measured by following the decay of the average volume of resolvable backbone ^1^H‐^15^N cross‐peaks of monomeric htt^ex1^Q_35_ in a set of serially acquired 2D ^1^H‐^15^N band‐selective optimized flip‐angle short‐transient heteronuclear multiple quantum coherence (SOFAST‐HMQC)^[^ ^52^, ^56^ ^]^ spectra (≈12 min per 2D spectrum). The average cross‐peak volume was obtained by averaging the volumes of all well‐resolved cross‐peaks in the 2D ^1^H‐^15^N correlation spectrum (residues 3, 5, 6, and 8–15 in the NT region, residues 50 and 51 at the end of the polyQ_35_ region, and residues 64, 71, 75, 92, 94, 96, and 98–100 in the PRD). The continuous line in the absence of SeNPs is the simulated decay curve using the values of the tetramerization equilibrium association constant (*K*_tet_ = 7.4 × 10^6^ M^−3^) and the rate constants for oligomer conversion (*k*_C_ = 0.07 h^−1^), elongation (*k*_+_ = 6.4 × 105 M^−1^ h^−1^), and first‐order secondary nucleation (*k*_S_ = 0.3 M^−1^ h^−1^) determined previously.^[^ ^27^ ^]^ The initial conditions are [*m*] = 300 μM, [*M*] = 0, and [*P*] = 0.1 nM. The continuous line in the presence of SeNPs represents the best fit obtained by optimizing the value of the apparent equilibrium dissociation constant for the binding of SeNPs to fibril ends ($K_{\text{diss}}^{P}$= 10.2 ± 2.1 nM) with the values of *K*_tet_, *k*_C_, *k*_+_, and *k*_S_ fixed to the values listed previously. The normalized *χ* ^2^ of the combined fit to the data in the absence and presence of SeNPs is 1.03. A virtually identical fit (normalized χ^2^ = 1.04), shown by the dashed line in the presence of SeNPs, is obtained if one assumes that inhibition of aggregation occurs by binding of prenucleation tetramers *T* to SeNPs ($K_{\text{diss}}^{T}$ = 8.2 ± 1.5 nM), thereby preventing conversion of *T* to *P* (see text, Section 2.3). However, the latter mechanism is inconsistent with DLS measurements (Figure S1C, Supporting Information), fluorescence immunostaining (Figure 3A), and electron microscopy (Figure 3B).

Huntington's disease is a fatal autosomal dominant neurodegenerative condition arising from triplet CAG nucleotide expansion within exon‐1 of the *HTT* gene encoding the huntingtin exon‐1 protein, htt^ex1^.^[^ ^1^, ^2^, ^3^ ^]^ Htt^ex1^ comprises three distinct regions: a 17‐residue amphiphilic N‐terminal (NT) domain, a polyglutamine repeat of variable length (Q_*n*_), and a proline‐rich domain (PRD). Huntington's disease develops when the polyQ repeat exceeds 35–36 residues to generate aggregation‐prone htt^ex1^ with consequent accumulation of fibrils^[^ ^4^, ^5^, ^6^ ^]^ that form intracellular neuronal inclusion bodies.^[^ ^7^ ^]^ Although an atomic resolution structure of htt^ex1^ fibrils has yet to be determined, solid‐state NMR spectroscopy,^[^ ^8^, ^9^, ^10^, ^11^ ^]^ electron paramagnetic resonance spectroscopy,^[^ ^12^ ^]^ cryoelectron tomography,^[^ ^13^, ^14^ ^]^ and cryoelectron microscopy^[^ ^15^, ^16^, ^17^ ^]^ have shown that the fibril core consists of a rigid β‐hairpin/sheet formed by the polyQ region, while NT helices of intermediate dynamics and highly mobile PRD domains are located on the outside of the fibrils. The rate of polyQ aggregation is modulated by the flanking NT and PRD regions with the former significantly enhancing fibril formation.^[^ ^18^, ^19^, ^20^, ^21^ ^]^ In a series of papers using exchange‐based NMR spectroscopy, we have shown that prenucleation tetramerization of the NT domain to form a *D*_2_ symmetric four‐helix bundle on the microsecond timescale^[^ ^22^, ^23^, ^24^ ^]^ increases the local concentration of the polyQ tracts and provides a template that is an essential prerequisite for much slower nucleation and fibril formation that occurs on the timescale of hours.^[^ ^24^, ^25^, ^26^, ^27^ ^]^ The scheme for the aggregation mechanism of htt^ex1^Q_35_ that quantitatively accounts for the time courses of aggregation observed by NMR is shown in **Figure** **1**A.^[^ ^26^ ^]^ Reversible prenucleation oligomerization from monomer (*m*) via dimer (*D*) to tetramer (*T*) on the microsecond timescale results in a low population of *T* which undergoes slow conformational rearrangement to elongation‐competent tetramers with extendable ends *P*—“primary nuclei” (note that the designation “nuclei” and “extendable fibril ends” are used interchangeably throughout the text).^[^ ^26^ ^]^ The latter undergo elongation by the addition of monomers to the ends *P* to form fibrils *M*, and further fibrillization occurs by secondary nucleation on the surface of fibrils (Figure 1A). The latter process has recently been observed directly using high‐speed atomic force microscopy.^[^ ^28^ ^]^ The *T* to *P* conversion process is expected to be slow since a specific register between the polyQ strands is likely required to form elongation‐active nuclei *P*. Although their structure is unknown, the extendable ends *P* may be speculated to comprise the polyQ core in a β‐sheet conformation. The process of conversion is then likely to be facilitated by interactions between the hydrophobic moieties of interdigitated glutamine sidechains.^[^ ^10^ ^]^

It has already been shown that targeting on‐pathway prenucleation tetramerization can inhibit htt^ex1^ fibril formation in several ways: 1) sequestration of htt^ex1^ monomer by binding, for example, to the chaperonin GroEL/Hsp60, dramatically reduces the concentration of tetramer, given the cubic dependence of the population of tetramer on monomer concentration;^[^ ^29^ ^]^ 2) modification (e.g., oxidation of the Met^7^ sidechain to a sulfoxide by TiO_2_ nanoparticles^[^ ^30^ ^]^ and post‐translational phosphorylation of Ser and/or Thr residues^[^ ^31^, ^32^, ^33^ ^]^) or mutation^[^ ^34^ ^]^ of residues within the NT domain can inhibit either dimer or tetramer formation; and 3) polyproline‐binding proteins, such as profilin and the Src homology 3 domain, bind to the PRD and allosterically block tetramerization either at the monomer ↔ dimer or dimer ↔ tetramer steps, respectively.^[^ ^24^, ^25^ ^]^ Recently, it has been shown that the chaperone Hsp104 inhibits amyloid Aβ42 fibril formation substoichiometrically (with respect to monomer) by tightly binding to the extendable ends of nuclei and growing fibrils.^[^ ^35^ ^]^ We therefore sought to determine whether it was also possible to inhibit htt^ex1^ aggregation by binding to extendable fibril ends.

Selenium nanoparticles (SeNP) have recently been shown to be neuroprotective in a transgenic Huntington's disease model of *Caenorhabditis elegans* and to inhibit aggregation of huntingtin proteins.^[^ ^36^, ^37^ ^]^ In addition, SeNPs have been suggested as potential therapeutic agents for Parkinson's^[^ ^38^, ^39^ ^]^ and Alzheimer's^[^ ^40^ ^]^ diseases. Here, we use NMR spectroscopy, dynamic light scattering (DLS), fluorescence immuno‐based light microscopy, and negative stain transmission electron microscopy (TEM) to probe the kinetic mechanism whereby SeNPs modulate the kinetics of aggregation of htt^ex1^ proteins using two constructs: a nonpathogenic construct comprising 7 glutamines, htt^ex1^Q_7_, that only aggregates on a timescale of weeks,^[^ ^24^ ^]^ and a pathogenic construct comprising 35 glutamines, htt^ex1^Q_35_, that forms fibrils over a period of hours.^[^ ^25^ ^]^ We show that SeNPs reduce the rate of fibril formation in a substoichiometric manner with respect to the concentration of htt^ex1^Q_35_ monomer by binding with nanomolar affinity to the ends of elongation‐competent species.

## 2 Results

### 2.1 Following Aggregation of htt^ex1^Q_35_ by NMR

The aggregation/fibrillization of htt^ex1^Q_35_ is easily followed by acquiring a series of 2D ^1^H‐^15^N correlation spectra and monitoring the disappearance of htt^ex1^Q_35_ monomer (≈11.5 kDa) by the reduction in ^1^H_N_/^15^N cross‐peaks (Figure 1B). The key premise of this analysis is that the decay in monomer concentration can be fully accounted for by the formation of NMR invisible aggregates,^[^ ^25^, ^26^, ^41^ ^]^ as discussed in detail in a recent review.^[^ ^41^ ^]^ At 5 °C in the absence of SeNPs, the half‐life (*t*_1/2_) for monomer decay at an initial concentration of htt^ex1^Q_35_ monomer of 300 μM is ≈15 h which is increased to ≈20 h in the presence of an 8 mg ml^−1^ (42 nM) suspension of SeNPs (Figure 1B). Characterization of the SeNPs by DLS in the absence and presence of htt^ex1^Q_7_ and htt^ex1^Q_35_ is provided in Figure S1, Supporting Information.

Although the concentration of SeNPs used here is high in terms of biological studies, our purpose is to elucidate the mechanism of action of SeNPs rather than defining an optimal therapeutic dose. Further, the concentration of htt^ex1^Q_35_ used here is also much higher than physiological levels to ensure good signal to noise for the NMR data so that a high concentration of SeNPs is necessarily required. Finally, the concentration of SeNPs employed represents a compromise between achieving a measurable and reproducible effect under our experimental conditions while maintaining good stability and solubility of the SeNP particles. We also note that SeNPs, even at concentrations as high as 20 mM, display minimal toxicity in a *Caenorhabditis elegans* model and do not induce cell death.^[^ ^36^ ^]^

### 2.2 Interaction of SeNPs with htt^ex1^Q_7_ Monomer Using Dark‐State Exchange Saturation Transfer (DEST)

**Figure 2:**
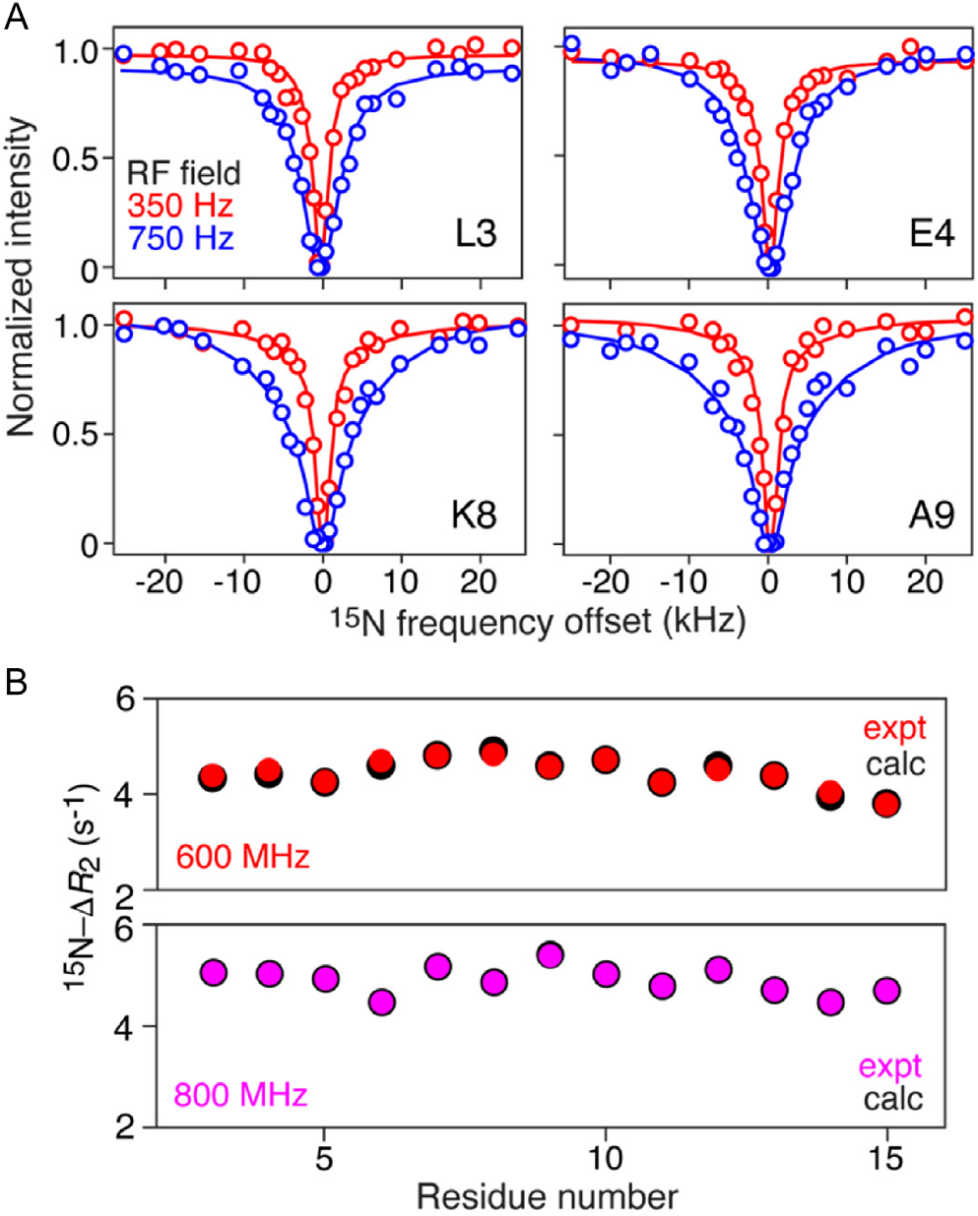
Interaction of 150 μM ^15^N‐labeled htt^ex1^Q_7_ monomers with 8 mg ml^−1^ (42 nM) SeNPs monitored by ^15^N‐DEST and ^15^N lifetime line broadening (Δ*R*_2_) NMR measurements. A) Examples ^15^N‐DEST profiles at two RF saturation fields (red, 350 Hz; blue, 750 Hz) applied for *T*_DEST_ = 700 ms. The experimental data, shown as circles, were recorded at a spectrometer frequency of 600 MHz, and the continuous lines represent the best fits using a two‐site exchange model. B) ^15^N‐Δ*R*_2_ measurements at 600 (top) and 800 (bottom) MHz spectrometer frequencies. The red and purple circles are the experimental data (and the errors are smaller than the circles), and the black circles are the best fits obtained using a two‐site exchange model. The ^15^N‐DEST and ^15^N‐Δ*R*_2_ data were fit globally.^[^ ^44^, ^45^ ^]^ The experimental data were recorded at 5 °C and pH 6.5. The complete ^15^N‐Δ*R*_2_ and ^15^N‐DEST datasets are provided in Figure S2 and S3, Supporting Information.

To explore the mechanism whereby SeNPs slow down the aggregation of htt^ex1^Q_35_, we first investigated the interaction of 150 μM ^15^N‐labeled htt^ex1^Q_7_ (≈7.4 kDa) with a suspension of 8 mg ml^−1^ (42 nM) SeNPs using ^15^N‐DEST and lifetime line broadening (^15^N‐Δ*R*_2_) NMR measurements (**Figure** **2**). Both NMR experiments rely on large differences in molecular weight between the NMR observable species (monomeric htt^ex1^Q_7_) and the “dark” state that is rendered “NMR‐invisible” due to very high molecular weight and, hence, very large ^15^N‐*R*_2_ transverse relaxation rates (htt^ex1^Q_7_ bound to SeNPs).^[^ ^42^, ^43^, ^44^ ^]^ Although htt^ex1^Q_7_ undergoes transient reversible tetramerization on the microsecond timescale, the population of tetramer is <0.1% under the experimental conditions employed, and htt^ex1^Q_7_ remains predominantly monomeric (>99.9%) over the time course of these experiments.^[^ ^24^ ^]^ The ^15^N‐Δ*R*_2_ data indicate that the interaction of htt^ex1^Q_7_ with SeNPs is limited to the NT region as evidenced by significant lifetime line broadening (Figure S2, Supporting Information). The PRD, in contrast, exhibits no lifetime line broadening (Figure S2, Supporting Information). Global fitting of the ^15^N‐DEST profiles (Figure 2A and S2, Supporting Information) and ^15^N‐Δ*R*_2_ data (Figure 2B) for the well‐resolved ^1^H‐^15^N cross‐peaks of the NT region to a two‐site exchange model^[^ ^45^ ^]^ yields a bound population *p*_B_ of ≈0.2%, with $k_{\text{on}}^{\text{app}}$ and *k*_off_ rate constants of 5.4 ± 0.1 and 2400 ± 100 s^−1^, respectively (see Experimental Section, Section 4.6 and 4.7, for details of the fitting procedure), corresponding to relatively weak binding with an apparent equilibrium dissociation constant *K*_diss_ (expressed in terms of SeNP concentration rather than SeNP sites) of ≈20 μM. The average ^15^N‐*R*_2_ value for the bound state is 11 570 ± 1400 s^−1^, consistent with an expected rotational correlation time of ≈12 μs for an SeNP particle with a diameter *d* ≈50 nm (Figure S1A, Supporting Information) (note that the value of ^15^N‐*R*_2_ is directly proportional to the rotational correlation time and the latter can be estimated independently from the particle diameter using the Stokes–Einstein relation).

From the value of *p*_B_, the concentration of bound htt^ex1^Q_7_ is ≈0.16 μM corresponding to a stoichiometry of ≈7 htt^ex1^Q_7_ molecules per SeNP particle which is compatible with the negligible increase of only ≈1 nm in particle diameter (Figure S1A,B, Supporting Information). However, the reduction in concentration of free monomer by sequestration on the surface of SeNPs is close to two orders of magnitude too small to account for the observed increase in the *t*_1/2_ for aggregation observed in Figure 1B.

### 2.3 Quantitative Analysis of Aggregation Kinetics of htt^ex1^Q_35_ in the Presence of SeNPs

To gain further insight into the mechanism of action of SeNPs, we proceeded to quantitatively analyze the aggregation kinetics of 300 μM htt^ex1^Q_35_ in the absence and presence of 8 mg ml^−1^ SeNPs at 5 °C (Figure 1B). We note that the premise of any rigorous kinetic modeling of inhibition is that the rate constants do not change upon addition of SeNPs. Changes in rate constants would mean that some “apparent” values are obtained as a result of the model not correctly taking correctly into account the interactions with inhibitor.

In the absence of SeNPs, there is excellent agreement between the experimental decay of htt^ex1^Q_35_ monomer as a function of time (Figure 1B, blue circles) and the simulated curve obtained using our previously published values for the tetramerization equilibrium association constant (*K*_tet_ = 7.4 × 10^6^ M^−3^) for htt^ex1^Q_35_ and the rate constants for oligomer conversion (*k*_C_ = 0.07 h^−1^), elongation (*k*_+_ = 6.4 × 10^5^ M^−1^ h^−1^), and first‐order secondary nucleation (*k*_S_ = 0.3 M^−1^ h^−1^), all obtained under the identical experimental conditions as in the current work.^[^ ^27^ ^]^ The decay of htt^ex1^Q_35_ monomer in the presence of SeNPs (Figure 1B, where the red circles are the experimental data and the continuous line the best fit) is then readily accounted for by nanomolar affinity binding of SeNPs to fibril ends *P* with an apparent equilibrium dissociation constant, $K_{\text{diss}}^{P}$, of 10.2 ± 2.1 nM (see Experimental Section, Section 4.7 for details of mathematical formulation). A virtually identical best fit (Figure 1B, dashed line) is also obtained with nanomolar binding of SeNPs to prenucleation tetramers *T* with an apparent equilibrium dissociation constant, $K_{\text{diss}}^{T}$, of 8.2 ± 1.5 nM. In this model, the binding of SeNPs to tetramers *T* prevents the conversion of the latter to the ends *P*. The exact value of $K_{\text{diss}}^{T}$ is, however, not meaningful, as it is very sensitive to errors in the starting concentration of htt^ex1^Q_35_ (a ≈2% error in *m*_tot_ leading to a ≈2‐fold change in $K_{\text{diss}}^{T}$). (A comparison of the simulated time courses for [*T*], [*P*], [*M*], and the [*M*]/[*P*] ratio in the presence and absence of SeNP for both models is provided in Figure S4 of the Supporting Information and shows very similar features for both models.) Further, we tested a model that allows for binding of SeNPs to tetramers *T* and nuclei *P* simultaneously with the same *K*_diss_. This model yielded a (common) *K*_diss_ value of 68 ± 5 nM, albeit with a ≈10% worse goodness‐of‐fit.

The average diameter of SeNPs upon completion of htt^ex1^Q_35_ aggregation/fibrillization is ≈35% larger (≈69 vs. ≈53 nm) and more heterogeneous (polydispersity index of 0.23 vs. 0.18) than the respective values for SeNPs in the presence of htt^ex1^Q_7_ at the same concentration (see DLS data in Figure S1C vs. Figure S1B, Supporting Information). Given that the values of *K*_tet_ (see Figure 1A) for transient prenucleation tetramerization of htt^ex1^Q_35_ and htt^ex1^Q_7_ are comparable (≈7 × 10^6^ M^−3^)^[^ ^23^, ^26^ ^]^ and therefore the concentration of tetramer in the two samples is similar, one can conclude that the increase in SeNP particle diameter and polydispersity in the presence of htt^ex1^Q_35_ is likely attributable to the binding of relatively large oligomeric species or large clusters of “converted” tetramers via their extendable ends *P*. Note that our minimalistic kinetic model does not rule out the appearance of higher‐order (>4) oligomers during the time course of fibril formation as long as such oligomeric structures are capable of elongation (i.e., formed from “converted” tetramers and have extendable ends *P*). In Section 2.4, we distinguish between all the possible binding modes of SeNPs using fluorescence immunostaining and electron microscopy.

### 2.4 Visualization of Interaction of SeNPs with Fibril Ends *P* by Fluorescence Immunosequester‐Based Spectroscopy and TEM

**Figure 3:**
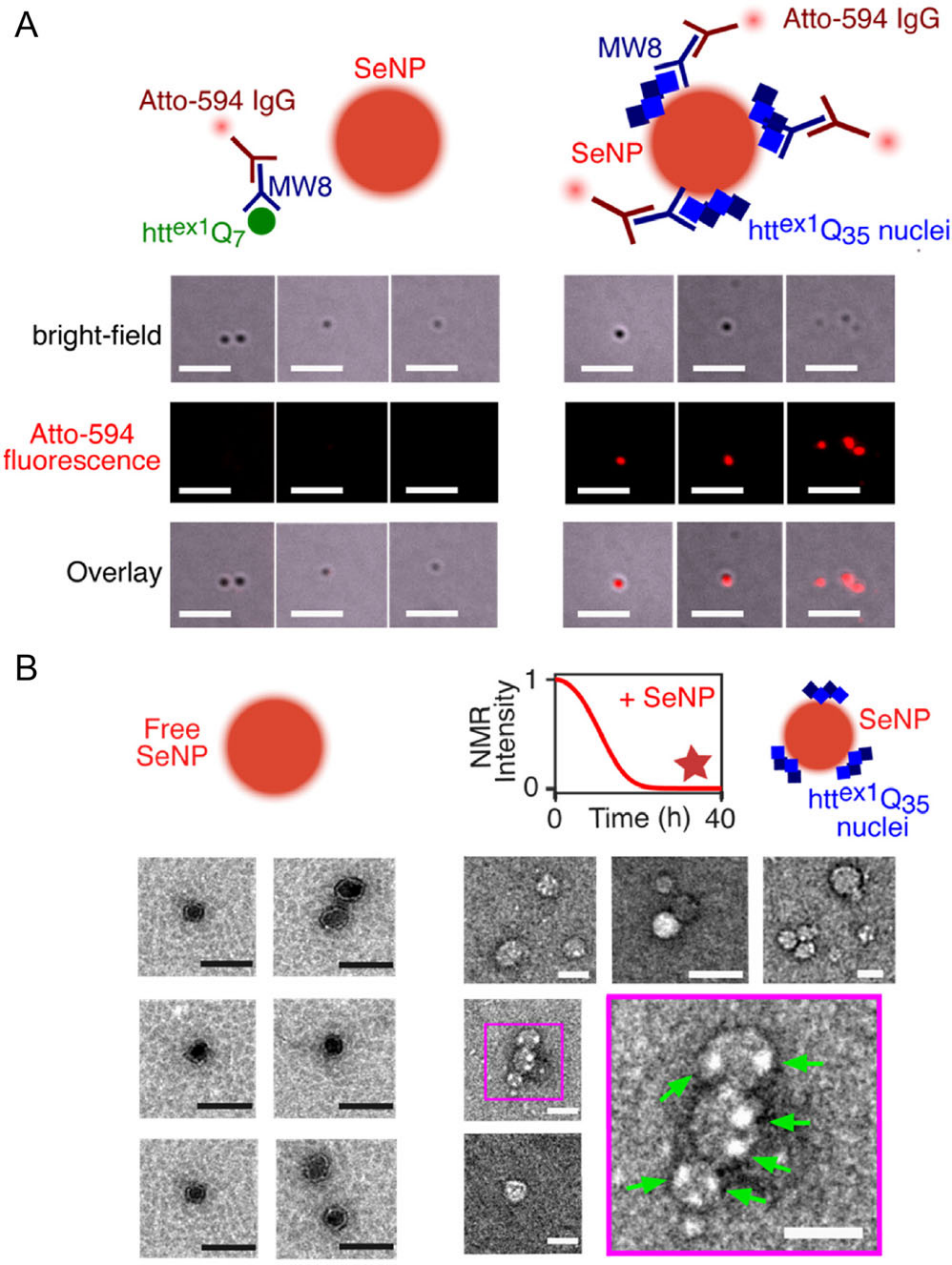
Characterization of the interaction of htt^ex1^Q_*n*_ (*n* = 7 and 35) with SeNPs using fluorescence immunostaining and TEM. A) Schematic of MW8 Mab/Atto‐594 IgG binding to htt^ex1^Q_7_ (left) and htt^ex1^Q_35_ (right) in the presence of SeNPs (top). Brightfield, Atto‐594 fluorescence and overlay images obtained with 300 μM htt^ex1^Q_7_ (left) or htt^ex1^Q_35_ (right) in the presence of 8 mg ml^−1^ SeNPs (bottom). Note the SeNPs were added to monomeric htt^ex1^Q_35_ and the images were obtained after 40 h incubation at which time htt^ex1^Q_35_ aggregation is complete. Scale bars: 5 μm. Control experiments in the presence of SeNPs with MW8 mAb/Atto‐594 IgG either alone or in the presence of added mature htt^ex1^Q_35_ fibrils are shown in Figure S5A,B, Supporting Information. B) Negative stain TEM images of empty SeNPs (left) and SeNPs incubated with htt^ex1^Q_35_ and visualized after 40 h upon completion of aggregation (right), as indicated by the star in the schematic of monomeric htt^ex1^Q_35_ decay as a function of time, display distinct morphologies. The patches of white on the surface of the SeNPs (indicated in the expanded view by the green arrows) arise from bound aggregation‐prone htt^ex1^Q_35_ extendable ends *P*. Scale bars: 50 nm.

To unequivocally establish that the interaction of the fibril ends *P*, rather than prenucleation tetramers, *T*, with SeNPs is responsible for slowing down the fibrillization of htt^ex1^Q_35_, we carried out fluorescence immuno‐based microscopy complemented by TEM. Using the MW8 primary monoclonal antibody (mAb),^[^ ^46^ ^]^ which specifically recognizes a 9‐amino acid epitope at the C‐terminal end of the PRD, and a secondary antibody labeled with the fluorescent dye Atto‐594 (which has a lower limit of detection in the 50–500 pM range), we first examined SeNPs incubated with 300 μM htt^ex1^Q_7_. The optical images showed no fluorescence colocalized with the SeNPs, indicating that neither htt^ex1^Q_7_ monomers or tetramers bind tightly to SeNPs (**Figure** **3**A, left panel). (Note that from the ^15^N‐DEST/ΔR_2_ data, the binding of htt^ex1^Q_7_ monomers to SeNPs is weak with a *K*_diss_ value of ≈20 μM, and exchange is sufficiently fast that any bound monomeric htt^ex1^Q_7_ would be removed during the washing steps of the immuno‐staining procedure.) In contrast, SeNPs incubated with 300 μM htt^ex1^Q_35_, imaged at 40 h after the complete disappearance of htt^ex1^Q_35_ monomer (monitored by NMR), display a strong fluorescent signal colocalized with the SeNPs (Figure 3A, right panel). No colocalization, however, is observed when mature htt^ex1^Q_35_ fibrils are incubated with SeNPs (Figure S5, Supporting Information). Taken together, these data indicate that SeNPs bind tightly to the extendable ends *P* of relatively large htt^ex1^Q_35_ oligomeric species, but not to the surface of mature fibrils of htt^ex1^Q_35_.

The above conclusions are also supported by the simulations of the time dependence of the species concentrations during the course of aggregation shown in Figure S4, Supporting Information. In the case of the kinetic model in which the extendable ends *P* bind to SeNPs, species with one extendable end bound to SeNPs ($P_{\text{bound}}^{1}$) reach an equilibrium value of ≈40 nM (Figure S4A, Supporting Information) and would therefore be detectable by immunofluorescence. In contrast, in the model in which the prenucleation tetramer *T* binds to SeNPs, all bound tetramer is gone after 40 h (Figure S4B, Supporting Information).

Negative staining TEM (Figure 3B) further supports the binding of extendable ends *P* to SeNPs and reveals that the surface of SeNPs is populated with large oligomeric species that appear as white patches. The latter are morphologically distinct from fibrils and correspond to aggregation‐prone extendable ends. Analysis of the brightest, well‐localized white patches from some of the TEM images provides length values ranging from ≈50 to ≈120 Å for the oligomers (Figure S6, Supporting Information). Assuming that the oligomeric core comprises a β‐sheet structure with each monomeric unit forming a β‐hairpin/sheet and an interstrand distance of 4.8 Å, one can deduce that the average number of monomeric units in the SeNP‐bound oligomers (at one extendable end) ranges from ≈12 to ≈25.

## 3 Conclusions

In summary, we have shown that elongation‐competent extendable ends or nuclei, *P*, of htt^ex1^Q_35_ bind with nanomolar affinity to the surface of SeNPs, thereby reducing the pool of free extendable ends *P* and slowing down subsequent fibrilization. Thus, SeNPs interact with htt^ex1^ proteins differently from other nanoparticles such as TiO_2_ nanoparticles that bind transiently to the NT domain and oxidize the Met^7^ side chain^[^ ^30^ ^]^ or lipid nanoparticles that sequester the htt^ex1^ monomer.^[^ ^47^ ^]^

It is important to note that the extendable ends *P* are structurally distinct from the prenucleation tetramer *T*. In the latter, the NT region forms a four‐helix bundle, while the polyQ and PRD regions remain disordered.^[^ ^22^ ^]^ The species with extendable ends *P* likely comprise the same or similar structure as mature fibrils with a rigid polyQ core consisting of repeated β‐hairpin/β‐sheets^[^ ^15^, ^16^, ^17^ ^]^ with partially mobile NT helices and highly mobile PRD domains located on the outside.^[^ ^8^, ^9^, ^10^, ^11^ ^]^ We hypothesize that the surface of SeNPs, due to their chemistry and surface charge, interacts preferentially with the extendable ends *P* which present appropriate electrostatic and hydrophobic moieties to mediate nanomolar binding.

Given that SeNPs can cross the blood–brain barrier,^[^ ^39^ ^]^ it may be possible that complexes of SeNPs with nuclei and elongation‐competent extendable ends of htt^ex1^ proteins may undergo cellular degradation via autophagy, as observed for other nanoparticles,^[^ ^48^, ^49^ ^]^ thereby offering a potential neuroprotective pathway that might potentially be exploited for the management of Huntington's disease. While the current work is not directly translatable to cellular systems, it provides a mechanistic basis for the rational design and targeted development of nanoparticles that could potentially reduce or inhibit aberrant htt^ex1^ aggregation.

## 4. Experimental Section

1

1.1

##### Preparation and Characterization of SeNPs

SeNPs were synthesized as described previously.^[^ ^50^ ^]^ Briefly, a 50 mM solution of ascorbic acid in a ratio 1:6 was added to a water solution of 100 mM of sodium selenite and 8 mg ml^−1^ bovine serum albumin (BSA). After dropwise addition of ascorbic acid to the sodium selenite/BSA solution, the mixture was stirred at 600 rpm for 1 h at 4 °C. The suspension was ultracentrifugated at 20 000 g for 15 min. and extensively washed with washing buffer (20 mM sodium phosphate, 50 mM NaCl, and pH 6.5) five times. The recovered SeNP pellet was then lyophilized and stored at −80 °C. The size distribution of the SeNP nanoparticles (at a concentration of 8 mg ml^−1^) was determined by DLS using a Zetasizer Nano ZS instrument (Malvern Instruments, UK) at 5 °C (Figure S1, Supporting Information). Each measurement consisted of 15 runs, and experiments were carried out in triplicate. Sample preparation and characterization were carried out in the dark. We calculated, based on the density of Se and mean diameter of the SeNPs, that a concentration of 8 mg ml^−1^ corresponded to a concentration of 42 nM.

##### Expression and Purification of htt^*ex1*^ *Q*_*7*_ *and htt* ^*ex1*^ *Q*_*35*_ *Peptides*

Uniformly, ^15^N‐labeled htt^ex1^Q_7_ and htt^ex1^Q_35_ were expressed, purified, and ^15^N‐labeled as described previously.^[^ ^27^ ^]^ All NMR samples were prepared by dissolving an aliquot of the lyophilized proteins in a 13.8 mM monobasic sodium phosphate buffer, pH 4.6, containing 50 mM NaCl in 10% D_2_O/90% H_2_O v/v. The pH of the buffer was subsequently adjusted to 6.5 by adding dibasic sodium phosphate for a final concentration of 20 mM. (Note that pH 6.5 is the optimal pH for solution NMR measurements to reduce exchange of amide protons with water that can result in line broadening.) The concentrations of htt^ex1^Q_7_ and htt^ex1^Q_35_ were determined from the UV absorbance at 205 nm.^[^ ^51^ ^]^ NMR measurements of htt^ex1^Q_35_ aggregation profiles in the absence and presence of SeNPs were started as soon as possible after sample preparation. We estimated that the average “dead time” from sample preparation to the start of NMR recording was on the order of 12–14 min. All NMR samples were placed in Shigemi tubes (sample volume ≈300 μL).

##### NMR Measurements

All NMR experiments were recorded at 5 °C on 600 and 800 MHz AVANCE HD (600) or NEO (800) Bruker NMR spectrometers equipped with TCI triple‐resonance *x*,*y*,*z*‐axis gradient cryogenic probes.

2D ^15^N‐DEST measurements^[^ ^42^ ^]^ on 150 μM htt^ex1^Q_7_ in the presence of 8 mg ml^−1^ SeNPs were performed at 600 MHz by applying a 0.7 s ^15^N continuous wave saturation pulse at radiofrequency (RF) field strengths of 350 and 750 Hz. The ^15^N carrier frequency offsets in kHz were −25, 20, 18, 15, 10, 7, 6, 5, 4, 3, 2, 1, 0.5, 0, −0.5, −1, −2, −3, −4, −5, −6, −7, −10, −15, −18, −20, and −25. All experiments were normalized to a reference experiment with the frequency offset set to −1 MHz.

^15^N‐Δ*R*_2_ values,^[^ ^42^ ^]^ given by the difference in ^15^N‐*R*_2_ values of 150 μM htt^ex1^Q_7_ obtained in the absence and presence of 8 mg ml^−1^ SeNPs, were determined as described previously.^[^ ^24^ ^]^^15^N‐*R*_2_ values were calculated from the measured ^15^N‐*R*_1ρ_ (using a spin‐lock RF field of 750 Hz) and ^15^N‐*R*_1_ values using the same procedures and pulse sequences as in our previous work.^[^ ^24^ ^]^

Aggregation/fibrillization of 300 μM ^15^N‐labeled htt^ex1^Q_35_ was monitored by following the disappearance of monomeric htt^ex1^Q_35_, quantified from the volumes for all resolvable ^1^H_N_‐^15^N cross‐peaks in 2D ^1^H‐^15^N SOFAST‐HMQC spectra^[^ ^52^ ^]^ recorded at 600 MHz. Each 2D ^1^H‐^15^N SOFAST‐HMQC spectrum was acquired using a recycle delay of 0.2 s and a total of 100* × 2048* complex data points in the indirect (^15^N) and direct (^1^H) dimensions, respectively, with respective acquisition times of 58 and 136 ms. The number of scans was set to 16, resulting in a total acquisition time of ≈12 min per 2D spectrum.

All NMR spectra were processed and analyzed using the NMRPipe/NMRDraw program^[^ ^53^ ^]^ and in‐house MATLAB/python scripts.

##### Fluorescence Microscopy

Fluorescence microscopy images were acquired using a Leica DM6000B microscope equipped with a 100×/1.40 oil objective. All the immuno‐based fluorescence samples were prepared as follows. SeNPs were conjugated with the primary mouse mAb MW8 (Sigma–Aldrich) after 40 h of incubation with either 300 μM htt^ex1^Q_7_ or htt^ex1^Q_35_. Note that htt^ex1^Q_7_ did not aggregate over the 40 h time period and therefore served as control for the presence or absence of prenucleation tetramer binding. In the htt^ex1^Q_35_, the 40 h time period corresponded to the end point of aggregation (Figure 1B). Following conjugation with the primary mAb (for ≈18 h at 4 °C), the SeNPs were conjugated with a secondary antimouse IgG antibody fluorescently labeled with the atto‐594 fluorophore. The SeNPs were then mounted with ProLong Gold Antifade (ThermoFisher). Slides were cured for 24 h at room temperature in the dark prior to observation. Controls of free SeNPs and SeNPs exposed to mature htt^ex1^Q_35_ fibrils were also acquired (Figure S5, Supporting Information). Fluorescence images were contrast adjusted using the ImageJ/Fiji software suite.

##### TEM

For TEM, 5 μL samples of completed aggregated/fibrillized htt^ex1^Q_35_ in the absence and presence of SeNPs were blotted onto carbon‐coated copper‐charged grids (3 to 4 nm carbon, Electron Microscopy Sciences) for 1 min, washed with water three times, and then stained with 2% uranyl acetate for 1 min. Images were recorded with a FEI Tecnai T12 electron microscope at 120 kV using a Gatan US1000 CCD camera.

##### Global Analysis of ^*15*^ *N‐DEST and* ^*15*^ *N‐ΔR*_*2*_ *Data*

The ^15^N‐DEST and ^15^N‐ΔR_2_ data obtained for the htt^ex1^Q_7_ in presence of SeNPs were globally fit to the function

(1) $$F=\alpha_{1}\sum_{i}{\left(\frac{\Delta R_{2,\text{obs}}^{i}-\Delta R_{2,\text{calc}}^{i}}{\sigma_{\Delta R_{2}}^{i}}\right)}^{2}+\alpha_{2}\sum_{i}\sum_{k}\sum_{j=1}^{3(2)}{\left(\frac{k_{\text{obs}}^{\text{i},\text{k},\text{j}}-k_{\text{calc}}^{\text{i},\text{k},\text{j}}}{\sigma_{K}^{i}}\right)}^{2}$$

where the first and second term relates to the Δ*R*_2_ data and DEST‐normalized intensities (*K*), respectively; the subscripts “obs” and “calc” refer to the experimental and calculated values; the superscripts *i*, *k*, and *j* correspond to residue number, DEST field strength, and DEST offset, respectively; and *α*_1_ and *α*_2_ are empirically determined weighting factors set to values of 2 and 1, respectively. The calculated DEST profiles were obtained using a closed‐form analytical solution for two‐site exchange.^[^ ^45^ ^]^ The calculated Δ*R*_2_ values were obtained by propagation of the appropriate set of Bloch–McConnell equations^[^ ^54^ ^]^ for two‐site exchange.^[^ ^44^ ^]^

##### Aggregation Kinetic Model and Global Fitting of Decays Monitored by Serially Acquired ^*1*^ *H‐* ^*15*^ *N SOFAST‐HMQC Spectra in the Absence and Presence of SeNPs*

In the absence of SeNPs, the kinetic model describing the formation of mature htt^ex1^Q_35_ fibrils was given by the following set of ordinary differential equations, as described previously^[^ ^26^ ^]^

(2) $$dP\left(t\right)/dt=k_{c}f\left[m\left(t\right)\right]+k_{s}m\left(t\right)M\left(t\right)$$

(3) $$dM\left(t\right)/dt=2k_{+}m\left(t\right)P\left(t\right)$$

where *m*(*t*), from material balance, is given by *m*(*t*) = *m*_tot_ – *M*(*t*) and f[m(t)] represents the time‐dependent concentration of “prenucleated” tetramers given by

(4) $$f[m(t)]=K_{\text{tet}}m{\left(t\right)}^{4}$$

where *k*_c_, *k*_+_, and *k*_s_ are the rate constants for unimolecular conversion of tetramer into nuclei *P*, elongation, and secondary nucleation, respectively, in units of h^−1^, M^−1^ h^−1^, and M^−1^ h^−1^. *K*_tet_ is the overall equilibrium association constant for tetramerization from monomer to tetramer given by (*K*_eq,1_)^2^ *K*_eq,2_ where *K*_eq,1_ and *K*_eq2_ are the equilibrium association constants for monomer to dimer and dimer to tetramer equilibria. Note that the optimal order of secondary nucleation with respect to monomer in this model is 1.

In the case of inhibitor *I* binding to fibril ends *P*, two coupled binding equilibria have to be considered

(5) $$P_{\text{free}}+I\overset{2k_{\text{on},1}}{\underset{k_{\text{off},1}}{⇌}}P_{\text{bound},1}$$

(6) $$P_{\text{bound},1}+I\overset{k_{\text{on},2}}{\underset{2k_{\text{off},2}}{⇌}}P_{\text{bound},2}$$

where *P*_free_ denotes the ends belonging to the fibrils free of inhibitor (unoccupied at both ends), *P*_bound,1_ and *P*_bound,2_ are the ends belonging to the fibrils bound to inhibitor at one and both ends, respectively, and (*k*_on,1_; *k*_on,2_) and (*k*_off,1_; *k*_off,2_) are association and dissociation rate constants of the corresponding binding events, respectively. The factors of 2 before *k*_on,1_ and *k*_off,2_ account for the multiplicity of binding sites: there are two ways for binding to occur to *P*_free_ and, likewise, two ways of dissociation from *P*_bound,2_. The equation describing the rate of fibril elongation is then given by

(7) $$\frac{dM\left(t\right)}{dt}=2k_{+}m\left(t\right)P_{f}\left(t\right)+k_{+}m\left(t\right)P_{b}^{*}\left(t\right)$$

where *P*_*f*_(*t*) and $P_{b}^{*}\left(t\right)$ are the number concentrations of *P*_free_ and *P*_bound,1_, respectively. Equation (7) reflects the fact that fibrils bound to inhibitor only at a single end (*P*_bound,1_) can still elongate from the free (unoccupied) end, while fibrils bound to inhibitor at both ends (*P*_bound,2_) cannot be elongated further.

Considering that the timescale of inhibitor binding events (μs to ms) is typically much faster than the timescale of aggregation (hours to days), direct incorporation of dynamic binding equilibria in Equations (5) and (6) into the equations of aggregation kinetics results in a set of very stiff differential equations. To avoid using assumptions about the rates of binding on the one hand and solving a set of very stiff differential equations on the other, we assumed that inhibitor binding occurs “infinitely” fast on the timescale of the aggregation process; namely, that the binding equilibrium is established effectively “instantaneously” at each sampling point of the aggregation profile.

When the inhibitor was present in large excess relative to *P* (total inhibitor concentration [*I*]_*T*_ » *P*), the following expressions may be used for the partitioning of the total concentration *P* onto the concentration of “free,” *P*_*f*_(*t*), “singly bound,”$P_{b}^{*}\left(t\right)$, and “doubly bound,” $P_{b}^{**}\left(t\right)$, parts

(8) $$P_{f}\left(t\right)=\frac{K_{D}^{2}}{{\left[I\right]}^{2}+2K_{D}\left[I\right]+K_{D}^{2}}P\left(t\right)$$

(9) $$P_{b}^{*}\left(t\right)=\frac{2K_{D}\left[I\right]}{{\left[I\right]}^{2}+2K_{D}\left[I\right]+K_{D}^{2}}P\left(t\right)$$

(10) $$P_{b}^{**}\left(t\right)=\frac{{\left[I\right]}^{2}}{{\left[I\right]}^{2}+2K_{D}\left[I\right]+K_{D}^{2}}P\left(t\right)$$

where the same equilibrium dissociation constant, *K*_*D*_ = *k*_off,1_/*k*_on,1_ = *k*_off,2_/*k*_on,2_, is assumed for inhibitor binding to the ends of free fibrils and singly bound fibrils (the two equilibria in Equations (5) and (6)). The *macroscopic* equilibrium constants for the two coupled equilibria in Equations (5) and (6) are related to each other through *K*_d,1_ = *P*_*f*_[*I*]/ $P_{b}^{*}$ = *K*_*D*_/2; *K*_d,2_ = $P_{b}^{*}$[*I*]/ $P_{b}^{**}$ = 2*K*_*D*_ and *K*_d,2_/*K*_d,1_ = 4.

When excess of inhibitor cannot be ensured, the concentration of free inhibitor [*I*] is expressed through $P_{b}^{*}$ and [*I*]_*T*_

(11) $$\left[I\right]=\frac{K_{D}\left({\left[I\right]}_{T}-P_{b}^{*}\right)}{K_{D}+P_{b}^{*}}$$

Solution of a quadratic equation for the fractional population of *P*_bound,1_, *p*_*b*_ =$P_{b}^{*}$/*P*

(12) $$\alpha {\left(p_{b}\right)}^{2}+\beta \left(p_{b}\right)+\gamma =0$$

where *α* = 2*P* ^2^, *β *= ([*I*]_*T*_ + *K*_*D*_)^2^ + 2* P*(*K*_*D*_ – [*I*]_*T*_), and *γ* = −2*K*_*D*_[*I*]_*T*_, yields $P_{b}^{*}=\left(\sqrt{\beta^{2}-4\alpha \gamma -\beta }\right)P/2\alpha$, and the concentration *P*_*f*_ and $P_{b}^{**}$can be calculated by inserting [*I*] obtained from Equation (11), into Equation (8) and (10), respectively.

The profiles of decay of the averaged normalized NMR signal intensities, *I*, measured at an initial concentration of 300 μM htt^ex1^Q_35_ monomer, *m*_tot_, were analyzed by minimization of the target function

(13) $$F=\sum_{i}\sum_{j}{\left[\frac{\left(I_{\text{exp}}-I_{\text{calc}}\right)}{\sigma }\right]}^{2}$$

where *I*_exp_ and *I*_calc_ are the experimental and calculated normalized intensities, respectively, *σ* is the standard error (assumed to be equal to 1/4^th^ of the standard deviation of the distributions of peak intensities for 25 residues of htt^ex1^Q_35_), the index *j* in the inner sum runs over all time‐points obtained, and the index *i* runs over all concentrations of added SeNPs. The normalized signal intensities *I*_calc_ were calculated from the relationship

(14) $$I_{\text{calc}}=A_{0}\left[1-\alpha^{\prime }M\left(t\right)/m_{\text{tot}}\right]$$

where *A*_0_ is a, global scaling factor that accounts for inaccuracies in the normalization of peak volumes (*A*_0_ typically optimizes to ≈0.99) and *α* is a scaling factor that takes into account the inaccuracies in the measurement of very low (close to zero) NMR peak volumes and/or some residual NMR signal intensities in the aggregated/oligomeric state(s). For both cases (in the absence and presence of SeNPs), the initial conditions were set the same: *P*(0) = 0.1 nM and *M*(0) = 0. Numerical integration was performed using an explicit Runge–Kutta formula implemented in the MATLAB “ode45” solver for nonstiff ordinary differential equations,^[^ ^55^ ^]^ with integration steps corresponding to the time‐points sampled in the NMR experiments. The global variable parameters in the minimization of the target function were *K*_D_ for the nuclei binding to SeNPs, while all the other equilibrium (*K*_tet_) and rate constants (*k*_c_, *k*_+_ and *k*_2_) were kept fixed to our previously published values.^[^ ^27^ ^]^ The uncertainties in the values of the optimized parameters, corresponding to confidence intervals of ±1 standard deviation, were determined from the variance–covariance matrix of the nonlinear fit. Uncertainties in the constants recalculated from the optimized parameters were determined by standard error propagation. All calculations were performed using an in‐house program written in MATLAB (MathWorks, Inc., MA).

## Supporting Information

Supporting Information is available from the Wiley Online Library https://doi.org/10.1002/smsc.202500345 or from the author.

## Conflict of Interest

The authors declare no conflict of interest.

## Author Contributions

**Francesco Torricella**: conceptualization (equal); formal analysis (equal); investigation (equal); methodology (equal); writing—original draft (equal). **Vitali Tugarinov**: formal analysis (equal); methodology (equal); writing—original draft (equal). **G. Marius Clore**: conceptualization (equal); formal analysis (equal); funding acquisition (lead); methodology (equal); supervision (lead); writing—original draft (equal); writing—review & editing (lead).

## Supporting information

Supplementary Material

## Data Availability

The data that support the findings of this study are available from the corresponding author upon reasonable request.
