# Supplementary material for: Kinetic Mechanism of Substoichiometric Inhibition of Huntingtin Exon‐1 Protein Aggregation by Selenium Nanoparticles
Source: Small Sci. 2025 Sep 13;5(11):2500345. doi: 10.1002/smsc.202500345 (PMC12622542; doi:10.1002/smsc.202500345)
Supplement: Supplementary file 1 — Supplementary Material [file SMSC-5-2500345-s001.pdf]

# SUPPORTING INFORMATION

## Kinetic mechanism of sub-stoichiometric inhibition of Huntingtin Exon-1 Protein Aggregation by Selenium Nanoparticles

**Francesco Torricella*,[a] Vitali Tugarinov and G. Marius Clore***

Laboratory of Chemical Physics, National Institute of Diabetes and Digestive and Kidney Diseases, National Institutes of Health, Bethesda, MD 20892-0520, United States.

[a]Present address: Instituto di Tecnologia, Brain and Development Disease, Via Morego 30, 16163 Gonova,Italy.

**Contents:**

- 6 SI figures

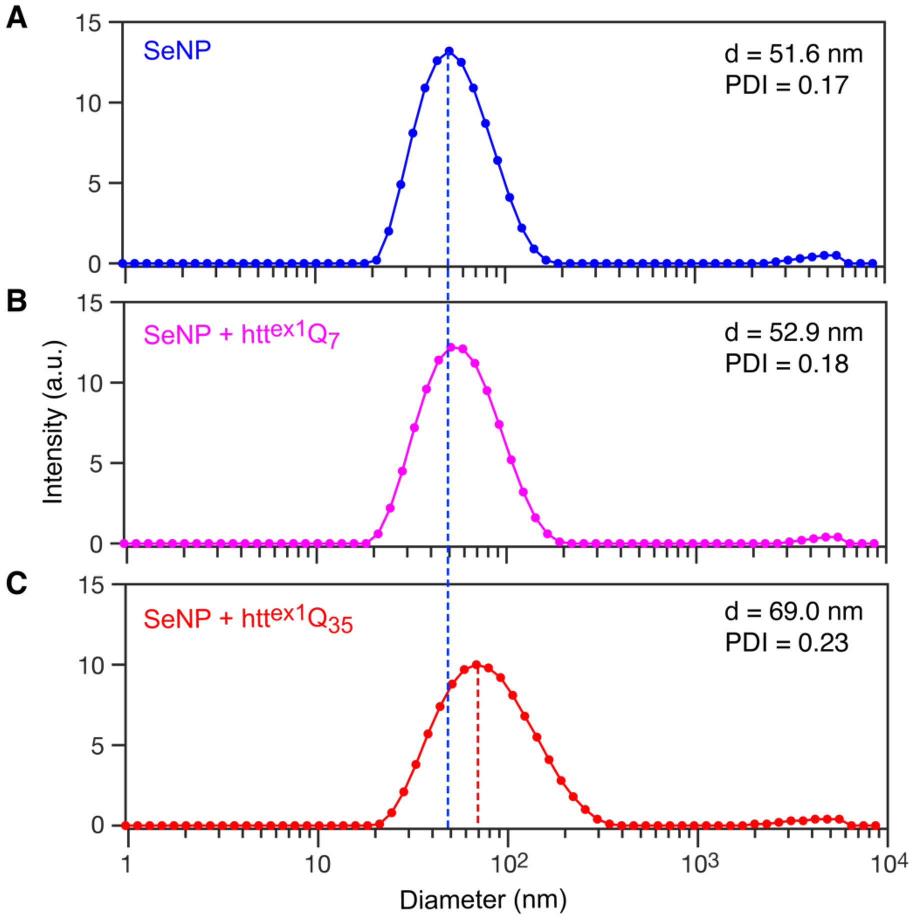


**Figure S1.** Characterization of SeNPs in the absence and presence of $htt^{ex1}Q_7$ or $htt^{ex1}Q_{35}$ by DLS. The plots show the intensity-weighted size distribution of SeNPs. Experiments were conducted on a suspension of 8 mg/ml SeNPs (A) alone and in presence of 300 μM (B) $htt^{ex1}Q_7$ or (C) $htt^{ex1}Q_{35}$ (after incubation for 40 h) at 5 °C using the same buffer used for the NMR experiments (50 mM NaCl, 20 mM sodium phosphate, pH 6.5, 90% $H_2O$/10% $D_2O$ v/v). The values for the *z*-average particle diameter (as derived from Cumulant analysis) and the polydispersity index (PDI) are indicated.

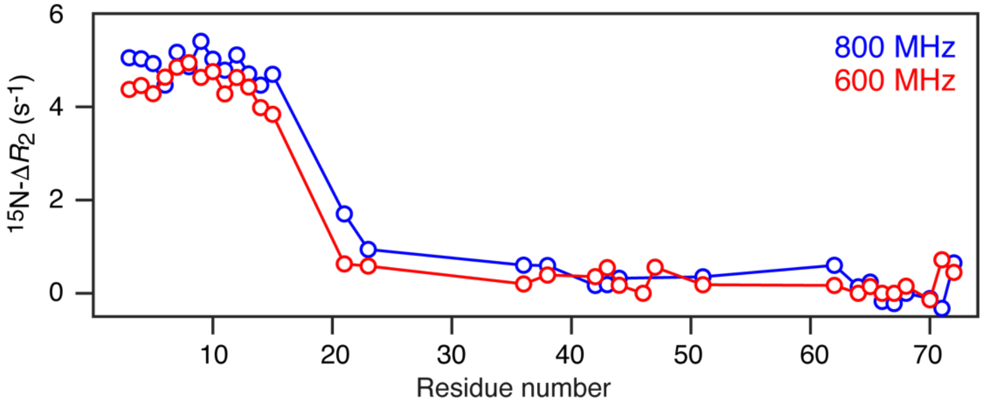


**Figure S2.** $^{15}N$ lifetime line broadening ($^{15}N$-$\Delta R_2$) of 150 μM htt$^{ex1}Q_7$ in the presence of 8 mg/ml SeNPs. The experimental NMR data, recorded at spectrometer frequencies of 600 and 800 MHz, were obtained at 5 °C and pH 6.5 in 50 mM NaCl, 20 mM sodium phosphate and 90% $H_2O$/10% $D_2O$ (v/v).

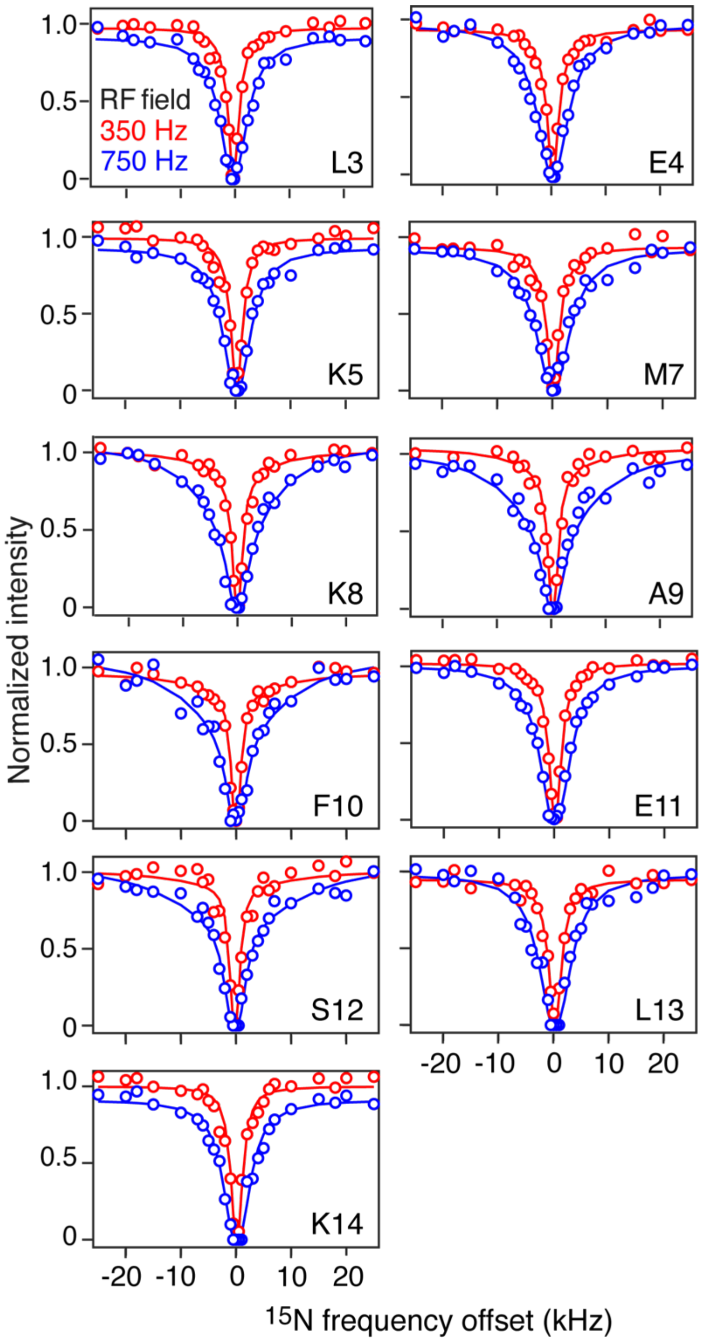


**Figure S3.** Complete set of $^{15}$N-DEST data recorded on 150 μM htt$^{ex1}$Q$_7$ in the presence of 8 mg/ml SeNPs at 5 °C. The experimental data are shown as circles and the continuous lines are the results of a global fit of the $^{15}$N-DEST and $\Delta R_2$ data to a two-site exchange model. The experimental data were recorded at a spectrometer frequency of 600 MHz. Sample conditions: 50 mM NaCl, 20 mM sodium phosphate, pH 6.5 and 90% $H_2O$/10% $D_2O$ v/v.

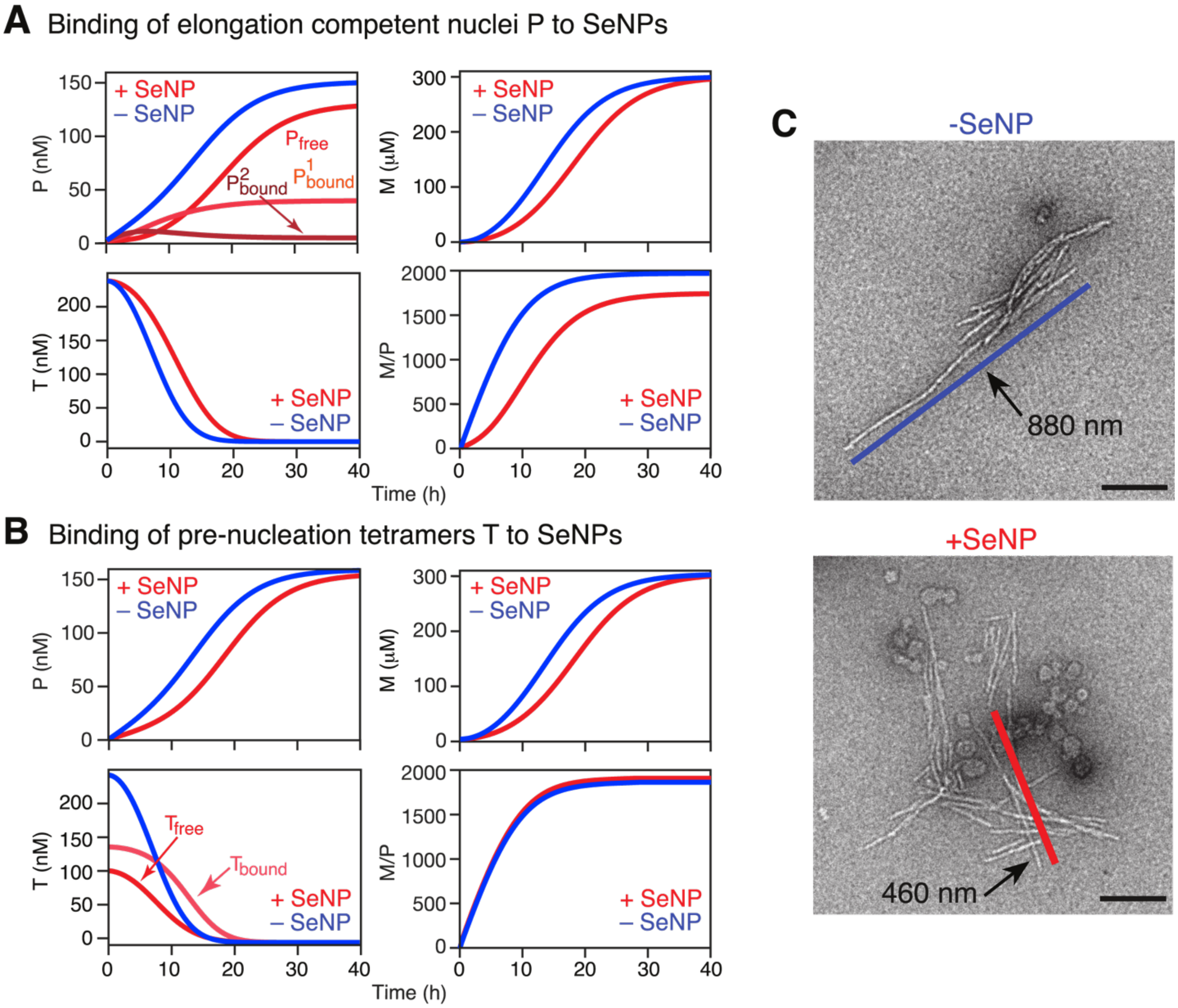


**Figure S4.** Simulated time courses for [P], [M], [T] and [M/P] using the optimized values of the equilibrium dissociation constants for the binding of SeNPs to either (A) nuclei P ($K_D^P$, = 10.2 ± 2.1 nM) or (B) pre-nucleation tetramers ($K_D^T$, = 8.2 ± 1.5 nM) obtained from fitting the decay of htt$^{ex1}$Q$_{35}$ monomer in the presence of SeNPs (see main text Fig. 1). The values for the tetramerization equilibrium association constant ($K_{tet}$ = 7.4 x $10^6$ $M^{-3}$) and the rate constants for oligomer conversion ($k_C$ = 0.07 $h^{-1}$), elongation ($k_+$ = 6.4 x $10^5$ $M^{-1}h^{-1}$) and secondary nucleation ($k_S$ = 0.3 $M^{-1}h^{-1}$) were fixed to the values determined previously in our laboratory.[2] (C) Negative stain transmission electron microscopy (TEM) images of htt$^{ex1}$Q$_{35}$ fibrils obtained in the absence (top) or presence (bottom, after 40 h incubation) of SeNPs. The maximum length of fibrils formed in the absence of SeNPs appears to be longer than those formed in the presence of SeNPs. This observation is consistent with the reduction in the M/P ratio (a measure of fibril length) seen in the model where SeNPs bind to *P* (A) rather than to *T* (B). Scale bars: 200 nm.

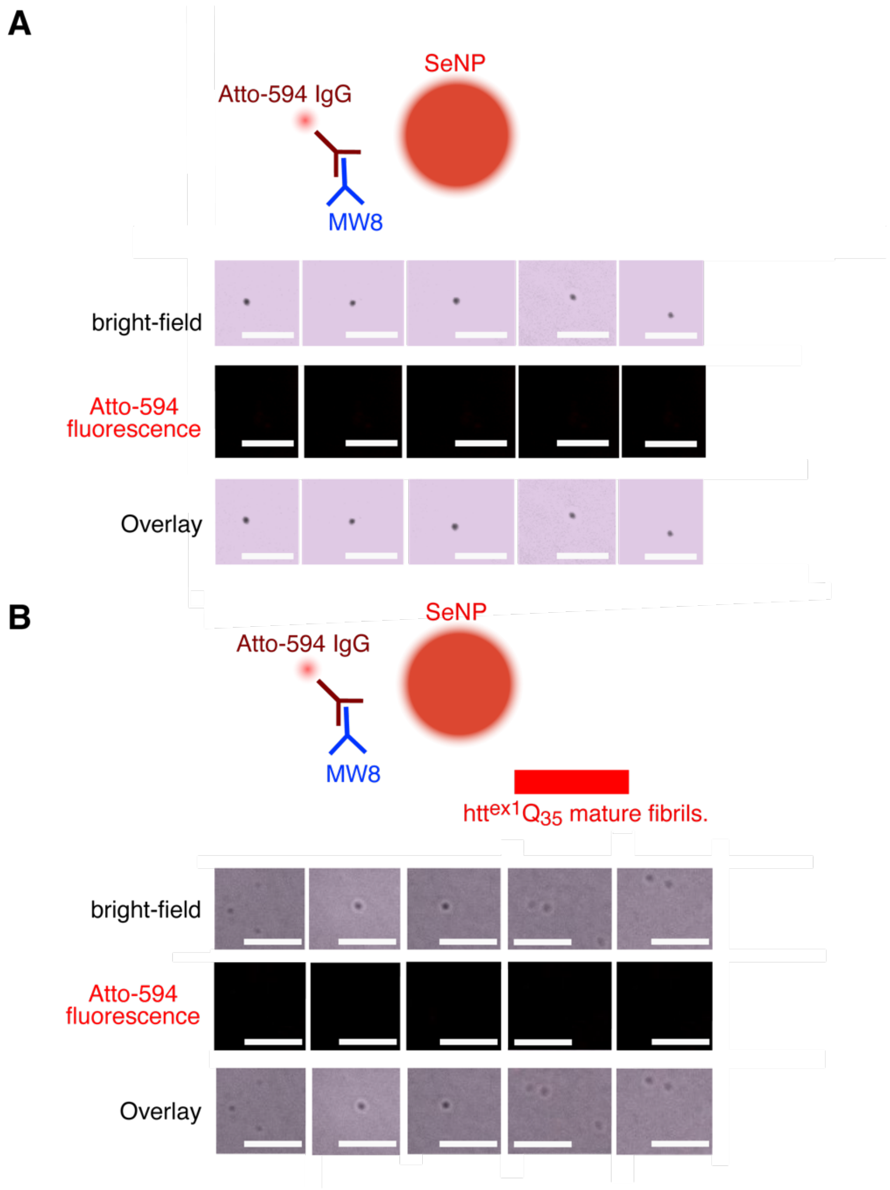


**Figure S5.** Control fluorescence immunostaining microscopy images obtained in the presence of SeNPs with the MW8 mAb and the Atto-594 fluorescently labeled secondary antibody (A) alone and (B) upon addition of mature htt$^{ex1}$Q$_{35}$ fibrils (obtained after complete fibrillization of a 300 μM monomeric solution of htt$^{ex1}$Q$_{35}$). No colocalization of fluorescence and the SeNPs is observed. Scale bars: 5 μm.

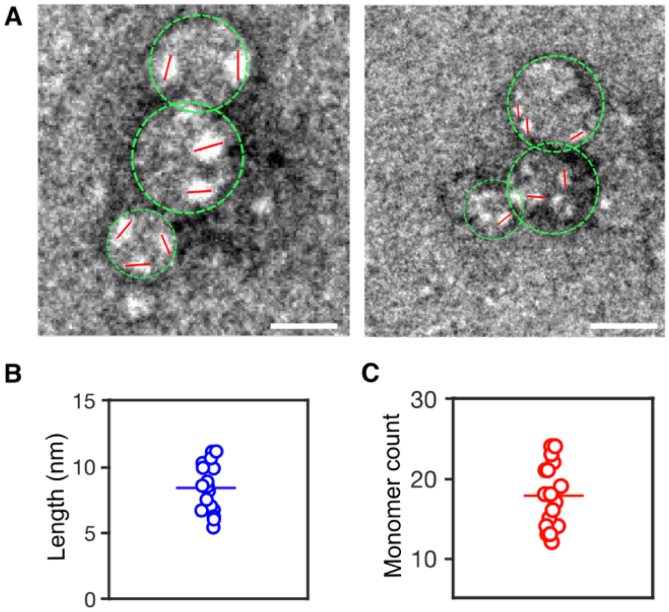


**Figure S6.** Morphological characterization of oligomeric htt$^{ex1}$Q$_{35}$ species bound to the surface of SeNPs. (A) Representative TEM micrographs showing SeNP-bound oligomers (green dashed circles) with individual bound segments highlighted (red lines) for length measurement. Scale bars: 50 nm. (B-C) Scatter plot of measured oligomer lengths (nm) and corresponding monomer unit counts estimated from the measured lengths assuming a distance of 4.8 Å between β-strands in the polyQ β-sheet core, with each monomer unit comprising a β-hairpin/sheet. In both plots the bar represents the mean value.
